# The ProkaBioDen database, a global database of benthic prokaryotic biomasses and densities in the marine realm

**DOI:** 10.1038/s41597-022-01281-x

**Published:** 2022-04-19

**Authors:** Tanja Stratmann

**Affiliations:** 1 Utrecht University, Department of Earth Sciences, Vening Meineszgebouw A, Princetonlaan 8a, 3584 CB Utrecht, The Netherlands; 2 HGF MPG Joint Research Group for Deep-Sea Ecology and Technology, Max Planck Institute for Marine Microbiology, Celsiusstr. 1, 28359 Bremen, Germany; 3 Department of Ocean Systems, NIOZ – Royal Netherlands Institute for Sea Research, PO Box 59, 1790 AB Den Burg (Texel), The Netherlands

**Keywords:** Marine biology, Archaeal biology, Marine microbiology

## Abstract

Benthic prokaryotes include Bacteria and Archaea and dominate densities of marine benthos. They play major roles in element cycles and heterotrophic, chemoautotrophic, and phototrophic carbon production. To understand how anthropogenic disturbances and climate change might affect these processes, better estimates of prokaryotic biomasses and densities are required. Hence, I developed the ProkaBioDen database, the largest open-access database of benthic prokaryotic biomasses and densities in marine surface sediments. In total, the database comprises 1,089 georeferenced benthic prokaryotic biomass and 1,875 density records extracted from 85 and 112 studies, respectively. I identified all references applying the procedures for systematic reviews and meta analyses and report prokaryotic biomasses as g C cm^−3^ sediment, g C g^−1^ sediment, and g C m^−2^. Density records are presented as cell cm^−3^ sediment, cell g^−1^ sediment/ sulfide/ vent precipitate, and cell m^−2^. This database should serve as reference to close sampling gaps in the future.

## Abstract (summary)

**Table:** 

Measurement(s)	prokaryotic benthic biomass • prokaryotic benthic density
Technology Type(s)	PLFA • microscopy • ATP
Sample Characteristic - Organism	unclassified Bacteria
Sample Characteristic - Environment	marine sediment • deep marine sediment • shallow marine sediment
Sample Characteristic - Location	Pacific Ocean • Atlantic Ocean • Indian Ocean • Southern Ocean • Arctic Ocean

## Background & Summary

Unicellular prokaryotes comprise the domains Bacteria and Archaea^1^. They have densities of 10^8^ to 10^9^ cells cm^−3^ ^2^ in the upper 10 cm of sediment and are therefore the most abundant benthic organisms. On a regional scale, prokaryotic densities in surface sediments decrease with depth (e.g.,^3^). Globally, however, their densities and biomasses do not decline with increasing water depth^4,5^. Prokaryotes are major players in the global cycling of elements, such as carbon^6–12^, nitrogen^13–15^, phosphorus^13,16,17^ and sulfur^18–21^, and they dominate sediment community oxygen consumption (SCOC) in deep-sea ecosystems >3,500 m water depth^22^.

Depending on the environmental conditions, prokaryotes can be involved in the transfer of organic matter to higher trophic levels: In the oxygen minimum zone of the Arabian Sea (Indian Ocean), the transfer of labelled carbon, that was taken up by prokaryotes, to their metazoan meiobenthic and macrobenthic consumers is relatively inefficient^6^. In comparison, for an intertidal area of the Scheldt estuary (North Sea), a model combined with a pulse-chase tracer experiment estimated that 3% of the prokaryotic carbon production was grazed upon by meiobenthos and 24% of this carbon production was consumed by macrobenthos^23^. In the deep-sea sediments of the Fram Strait (N Atlantic) and of the Clarion-Clipperton Fracture Zone (equatorial Pacific), however, no direct transfer of labelled carbon from prokaryotes to metazoan meiobenthos or metazoan macrobenthos was detected^8,24^.

Bacteria in marine surface sediments, i.e., sediment layers ranging from the sediment surface to – depending on the study – approximately 5 cm to 20 cm depth, contribute between 30 and 70% to total prokaryotic densities^25–28^. This corresponds to about 10^29^ bacteria cells living on our planet (uncertainty: 10-fold)^29^, of which 3.5 × 10^28^ ± 0.9 × 10^28^ occur in deep-sea surface sediments^4^. Hence, the upper 50 cm of said sediment are estimated to contain 1.29 Pg C^4^ (1 Pg = 1 petagram = 10^15^g) bacterial carbon which is up to 99% of the total estimated marine bacterial biomass (1.30 Pg C, uncertainty: 10-fold^29^). Bacteria can alter their environment, such as benthic cyanobacteria that can form so-called “microbially induced sedimentary structures”^30^. Particularly long, filamentous bacteria, known as “cable bacteria”, are even able to conduct long-distance electron transport over several centimeters^31–35^.

Archaea, whose most abundant phyla in the deep sea are Thaumarchaeota (53% of total Archaea density) and Euryarcheota (29% of Archaea density)^36^, account for <1 to 40% of prokaryotic densities in surface sediments^25,27,37^. Our planet is estimated to host 10^28^ marine benthic Archaea cells which is equivalent to 0.3 Pg C Archaea carbon (uncertainty: 13-fold)^29^.

Due to the prominent role of prokaryotes in the global carbon cycle, detailed knowledge about their biomasses and densities are necessary to understand how these microorganisms will be impacted by climate change and anthropogenic disturbances. Therefore, I prepared the open access “ProkaBioDen database”^38^ that, in comparison to preceding databases by Wei *et al*.^39^ and Rex *et al*.^5^, allows direct and free access to the data and transparently reports the selection process. It also covers the whole globe and not only the Atlantic Ocean and the Mediterranean Sea like in Danovaro *et al*.^36^ or is limited to specific water depths like in Danovaro *et al*.^4^.

The “ProkaBioDen database” lists 1,299 benthic prokaryotic biomass and 1,104 benthic prokaryotic density studies that were identified applying procedures for systematic reviews and meta analyses^40^. Based on this compilation, I extracted 1,089 georeferenced benthic prokaryotic biomass records and 1,875 georeferenced benthic prokaryotic density records from 85 and 112 chosen studies, respectively. I present benthic prokaryotic biomasses as g C cm^−3^ wet sediment, g C g^−1^ dry sediment, g C g^−1^ wet sediment, g C m^−2^ and benthic prokaryotic densities as cell cm^−3^ dry sediment, cell cm^−3^ wet sediment, cell g^−1^ dry sediment, cell g^−1^ dry sulfide, cell g^−1^ vent precipitate, cell g^−1^ wet sediment, cell m^−2^. All data further contain information about substrate type (e.g. *Calyptogena* sp. field, hydrothermal vent precipitate, iron oxidizing mat, mangrove, microbial mat, pogonophoran field, salt marsh, seagrass bed, sediment, sulfide chimney, sulfide oxidizing mat, sulfur band) and the methods applied to determine prokaryotic biomasses and densities and how researchers differentiated between Bacteria and Archaea densities. In this way, scientists can focus specifically on Archaea or Bacteria if they wish. The database is the first systematic open-access compilation of benthic Archaea and Bacteria densities and prokaryotic biomasses and densities and points towards undersampled geographic locations and water depth.

## Methods

**Fig. 1:**
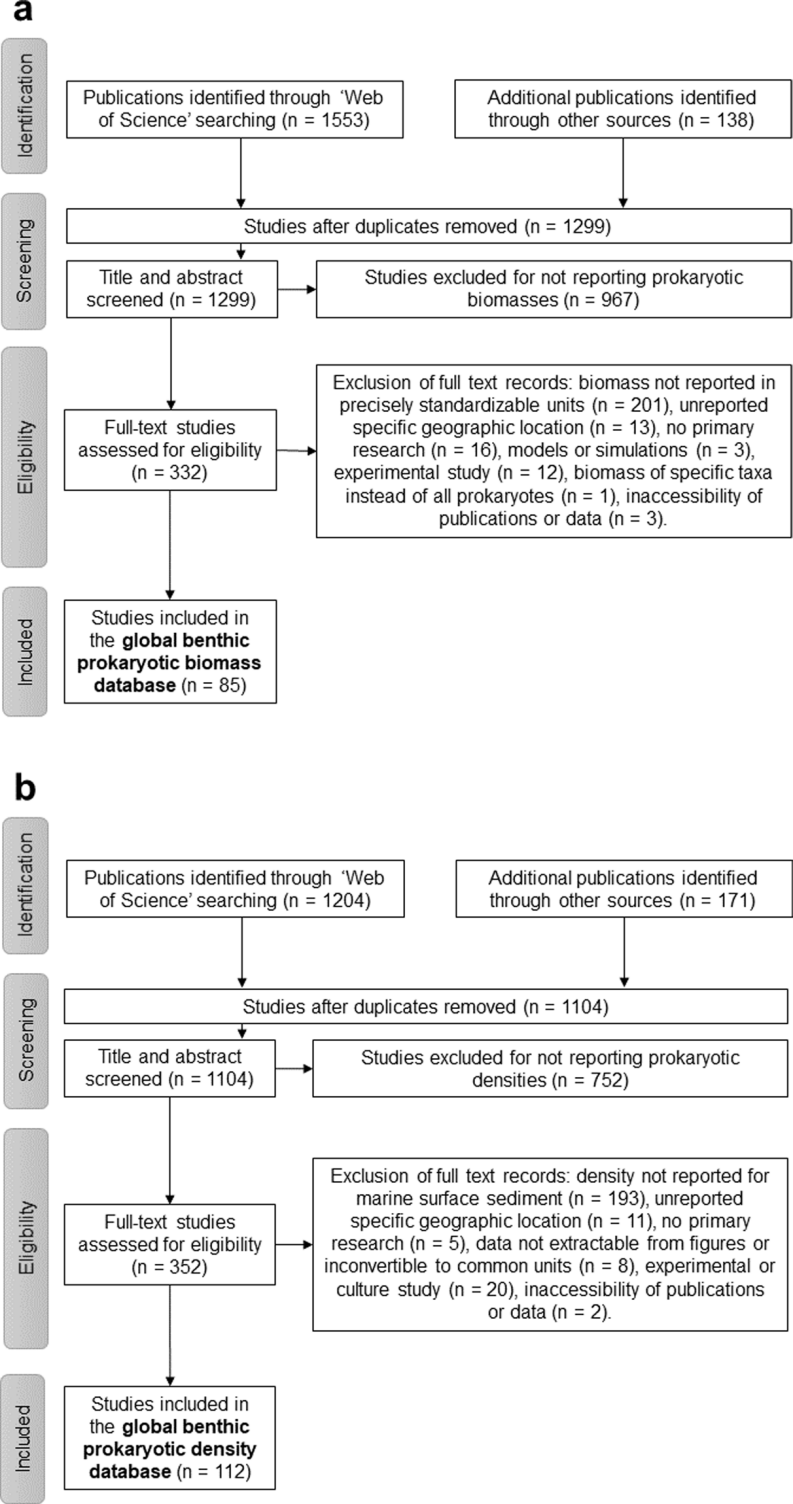
Flow chart describing how the database was created. It explains how studies were identified and why specific datasets were excluded from the final “ProkaBio” part (panel a) and from the final “ProkaDen” part (panel b) of the “ProkaBioDen database” following pre-defined selection criteria.

In March and June 2020, I compiled the “ProkaBio” part of the “ProkaBioDen database” applying the principles of “Preferred Reporting Items for Systematic Reviews and Meta-Analyses” (PRISMA)^40^. In the so-called “Identification” step, I identified 1,553 peer-reviewed articles in the *Web of Science* by using the key words “microb* biomass benth*”, “benthic prokaryotic biomass”, “benth* bacteria* biomass marin*”, and “Archaea biomass marin*”. Additionally, I found 138 publications in other sources, such as PANGAEA® Data Publisher (https://www.pangaea.de/) and peer-reviewed publications known to the author. After removing duplicate publications, I screened all titles and abstracts of 1,299 studies (Table 1; Fig. 1a; “Screening” step) and excluded 967 studies that did not report prokaryotic biomasses. In step 3, the so-called “Eligibility” step, I excluded in total 249 studies because they did not present prokaryotic biomasses in the marine sediment surface in standardizable units, i.e., in g C cm^−3^ wet sediment, g C g^−1^ wet sediment, g C g^−1^ dry sediment, or g C m^−2^. Furthermore, several studies lacked detailed geographical information about sampling stations or did not present primary research. Additional reasons for study exclusion were presenting prokaryotic biomasses for specific taxa instead of for all prokaryotes, being inaccessible, or introducing modelling, simulation, or experimental studies. In the final step, I included 85 studies from which I extracted 1,098 georeferenced benthic prokaryotic biomass records (Table 1, Fig. 1a).

**Table 1:** Specification of the ProkaBioDen database with file locations.

Source	Document name	Number of studies (records)	Data description	Method
10.5061/dryad.wm37pvmnv	List of studies for ProkaBio database	1,299	Alphabetical list of all references of studies about prokaryotic biomass that were identified when following the PRISMA Statement. Furthermore, it is indicated which studies were excluded during the screening processes and the eligibility check.	Literature search
10.5061/dryad.wm37pvmnv	List of studies for ProkaDen database	1,104	Alphabetical list of all references of studies about prokaryotic densities that were identified when following the PRISMA Statement. Furthermore, it is indicated which studies were excluded during the screening processes and the eligibility check.	Literature search
10.5061/dryad.wm37pvmnv	ProkaBio database	85 (1,098)	All prokaryotic biomass records compiled in the ProkaBio database.	Extraction of prokaryotic biomass records from the literature.
10.5061/dryad.wm37pvmnv	ProkaDen database	112 (1,875)	All prokaryotic density records compiled in the ProkaDen database.	Extraction of prokaryotic density records from the literature

In March and June 2020, I established the “ProkaDen” part of the database that consists of records of prokaryotic density as well as of density of Bacteria and of Archaea. Following the PRISMA approach^40^, I searched the *Web of Science* using the key words “marin* microb* abundance benth*”, “‘benthic bacteria’ abundance marin*”, “prokaryotic abundance marin*”, “prokaryotic density marin*”, “Archaea density abundance marin*”, “Archaea density marin*”, “Archaea abundance marin* benth*”, “Crenarchaea density abundance marin*”, “Crenarchaea density marin*”, “Crenarchaea abundance marin* benth*”, “Euryarchaea density abundance marin*”, and “Euryarchaea abundance marin* benth*” and found 1,204 peer-reviewed articles (Fig. 1b). I was aware of 171 additional studies that I included in the dataset which contained 1,104 studies after removing duplicates. In step 2 of the PRISMA approach (“Screening” step), I excluded 752 studies because they did not report benthic prokaryotic densities. In the “Eligibility” step, I furthermore omitted 239 studies because they did not present surface sediment prokaryotic densities or densities of a reduced number of prokaryotic taxa instead of reporting densities of all prokaryotes. I also removed studies that showed prokaryotic densities in poor-quality figures impeding data extraction and studies that listed densities which could not be converted to the common density units cell cm^−3^ dry sediment, cell cm^−3^ wet sediment, cell g^−1^ dry sediment, cell g^−1^ dry sulfide, cell g^−1^ vent precipitate, cell g^−1^ wet sediment, or cell m^−2^. I also excluded studies that reported experimental or culture studies and publications that I could not access. In the last step, I included 112 studies in the global benthic prokaryotic density database from which I extracted 1,875 georeferenced benthic prokaryotic density records (Table 1, Fig. 1a).

In 51% of the prokaryotic biomass studies and 34% of the prokaryotic density studies, the authors of the original publications did not report exact geographical coordinates (latitude, longitude) of the sampling stations. In these cases, I approximated the sampling locations using *Google Maps* based on maps from the original publications and indicated this with the label “approximated location”.

Prokaryotic biomasses were often not directly measured, but determined by extraction of bacterial adenosine triphosphate (ATP), extraction of bacterial phospholipid-derived fatty acid (PLFA), or by measuring prokaryotic densities. Subsequently, the authors of the original publications converted these data to prokaryotic biomasses using conversion factors (Table 2).

**Table 2:** References of biomass conversion factors to calculate prokaryotic biomass from prokaryotic densities measured with epifluorescence microscopy or with laser confocal scanning microscopy, from phospholipid-derived fatty acid (PLFA) concentrations, and from adenosine triphosphate (ATP) concentrations.

Biomass conversion from	Reference of conversion factor
Prokaryotic densities measured with epifluorescence microscopy	^44–65^
Prokaryotic densities measured with laser confocal scanning microscopy	^65^
PLFA concentrations	^12,66–70^
ATP concentrations	^71,72^

For cases where the prokaryotic biomasses and densities were not reported in the text or in tables, but were shown in figures, I extracted the data using ImageJ^41^.

## Data Records

The “ProkaBioDen database” is an open access database in the *Dryad Digital Repository* and contains two txt.files, i.e., the *List of studies for ProkaBio database* and the *List of studies for ProkaDen database*, and two xlsx.files, i.e., the file *ProkaBio database* and the file *ProkaDen database*^38^. The *List of studies* files report all studies in alphabetical order (prokaryotic biomasses: 1,300 studies:, prokaryotic densities: 1,104 studies) that I identified in the “Identification” step of the systematic review after I eliminated duplicates. Each data entry in the “ProkaBioDen database” includes information about the region and the ocean where the samples were taken, the geographical location (latitude, longitude), the water depth (in m), and the depth range after Dunne *et al*.^42^. The authors of said study classified the ocean into near-shore areas from 0 to 50 m water depth, continental shelves from >50 to 200 m water depth, continental slopes from >200 to 2,000 m water depth, and continental rises and abyssal plains >2,000 m depth. The database includes biomass and density records for individual sediment layers and information about the thickness of said sediment layers and its specific upper and lower boundaries when a layer was sliced horizontally, but also biomass and density records for vertically integrated sediment profiles. Additionally, the database contains information about sediment type, median sediment grain size (µm), sediment density (g cm^−3^), and porosity, and whether prokaryotic densities were reported for total prokaryotes, Bacteria, or Archaea.

## Technical Validation

**Fig. 2:**
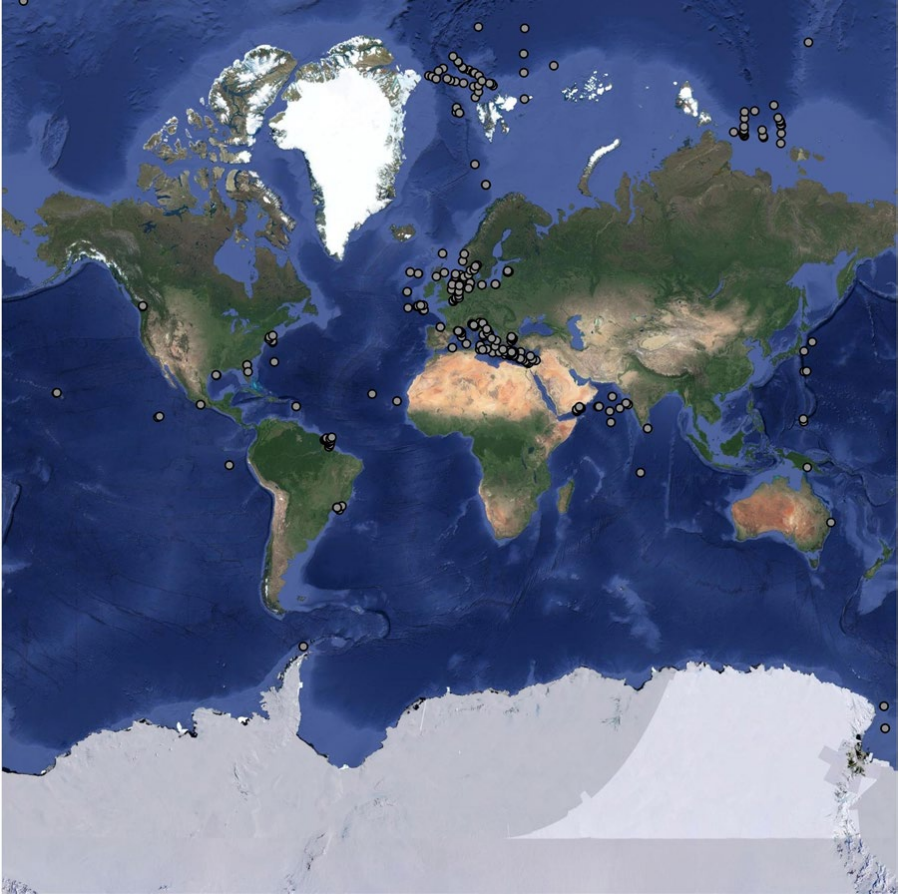
Global distribution of stations where benthic prokaryotic biomass samples were taken.

**Fig. 3:**
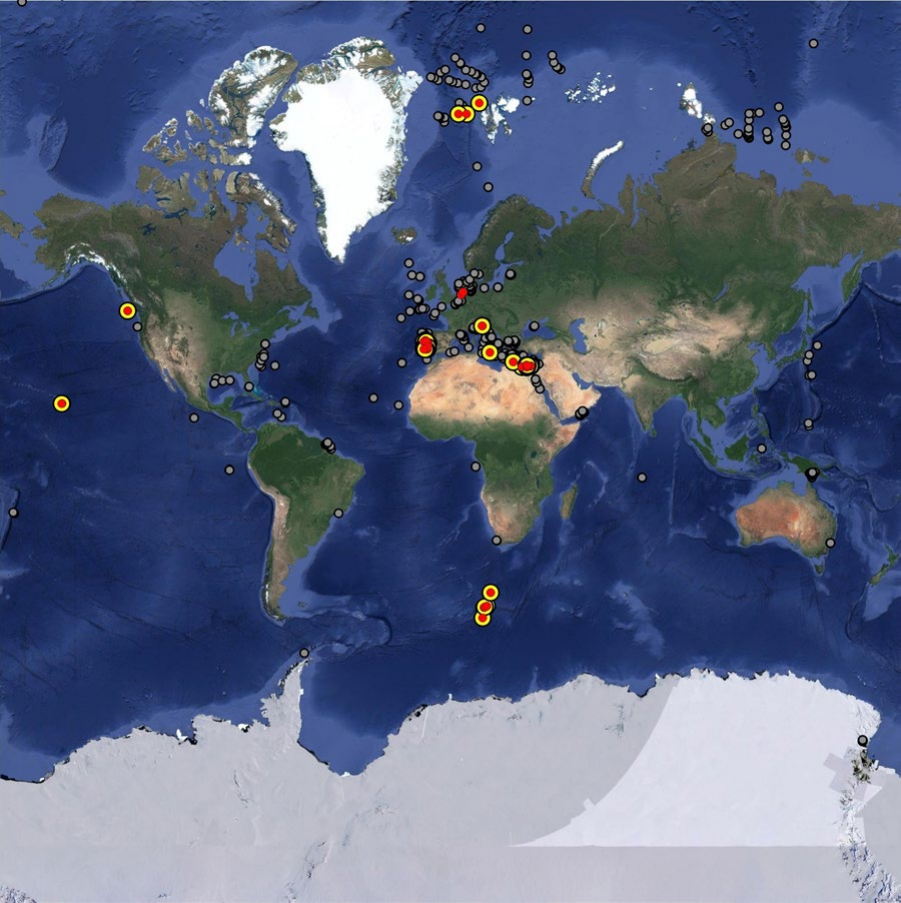
Global distribution of stations where benthic prokaryotic density (prokaryotes, Archaea, Bacteria) were taken. Color code: grey = prokaryotes, red = Bacteria, yellow = Archaea.

**Fig. 4:**
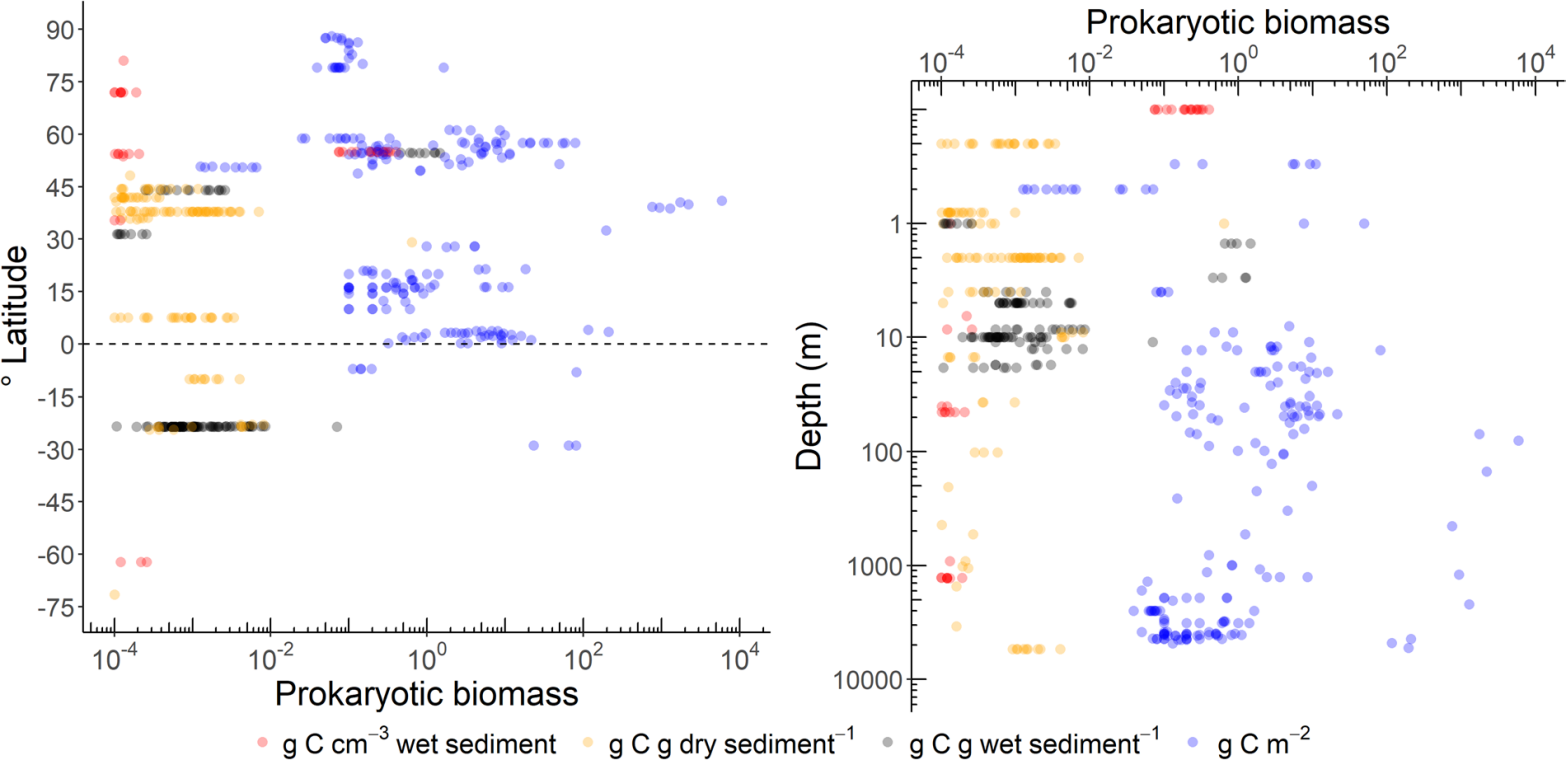
Benthic prokaryotic biomasses (left panel) along a latitudinal gradient with the equator indicated as dashed line and (right panel) along a water depth gradient (in m). Notice the logarithmic scale on the x-axis for the left panel and on the x- and y-axis for the right panel.

**Fig. 5:**
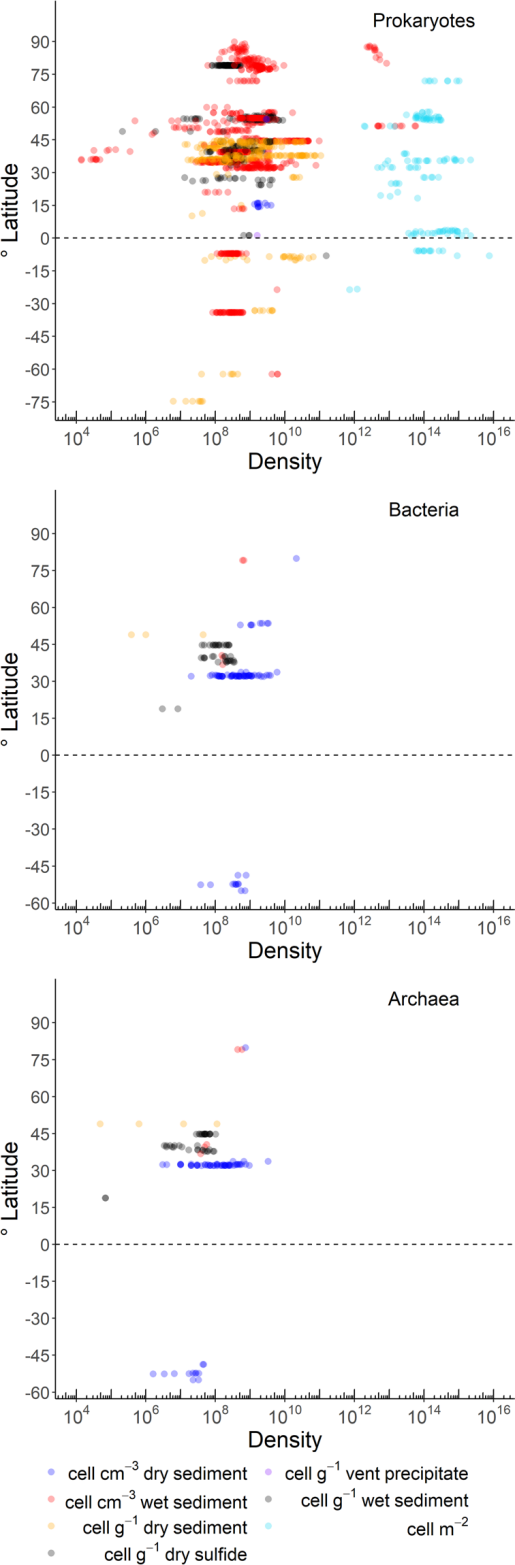
Benthic prokaryotic densities of total prokaryotes (upper panel), Bacteria (middle panel), and Archaea (lower panel) along a latitudinal gradient. The dashed line symbolizes the equator.

In the database, 40% of the benthic prokaryotic biomass samples originated in the Mediterranean Sea, 34% in the Atlantic, and 11% in the Arctic Ocean (Fig. 2). Most benthic prokaryotic density samples were taken in the Mediterranean Sea (42%), the Atlantic (27%), and the Arctic Ocean (15%), and also benthic Bacteria and Archaea densities were mainly sampled in the Mediterranean Sea (Bacteria: 62%, Archaea: 65%) and the Atlantic Ocean (Bacteria: 15%, Archaea: 17%) (Fig. 3). Both, benthic prokaryotic biomasses and densities were predominantly sampled in the northern hemisphere north of 1°N (biomass: 87%, density: 90%), whereas the southern hemisphere was seriously undersampled (Fig. 4 left panel and Fig. 5). Almost no samples were collected in the Indian Ocean (biomass: 7%, density: 1%) and the Southern Ocean (biomass: 2%, density: 1%). Hence, benthic prokaryote samples are biased towards the northern hemisphere and particularly towards the Mediterranean Sea and the North Atlantic.

**Fig. 6:**
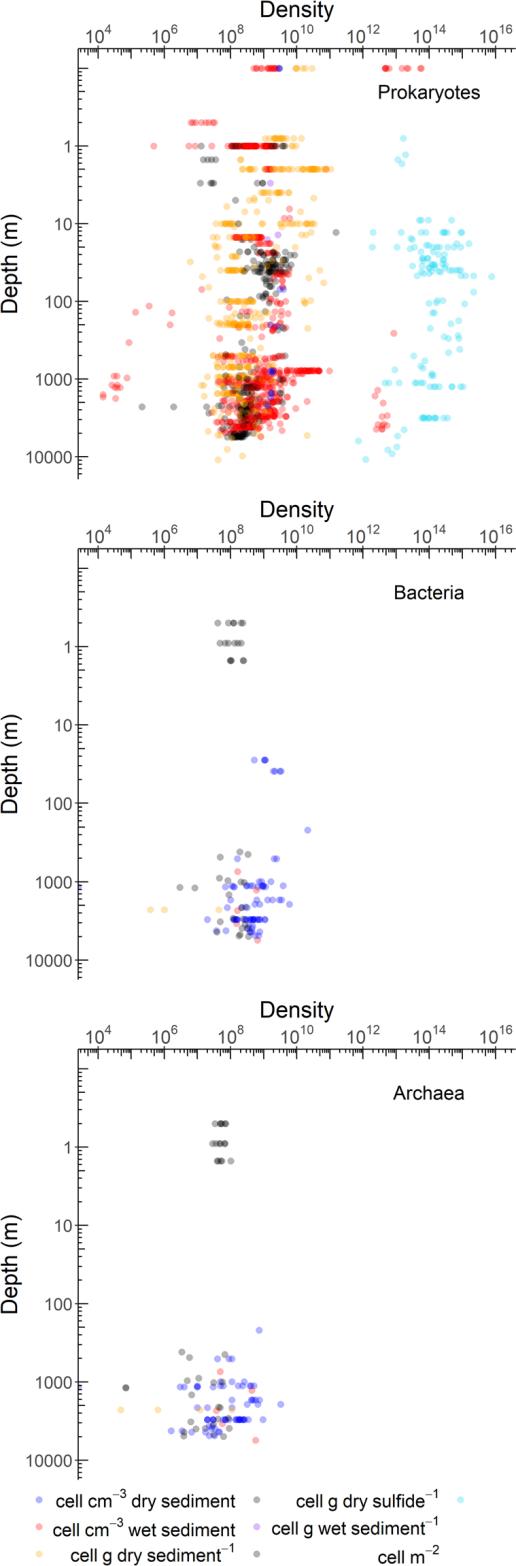
Benthic prokaryotic densities of total prokaryotes (upper panel), Bacteria (middle panel), and Archaea (lower panel) along a depth gradient (m).

Benthic prokaryotic biomasses were mostly quantified in the near-shore areas at <50 m water depth (54% of all samples, Fig. 4 right panel) that encompass 2% of the global ocean floor^42^. In comparison, only 15% of all benthic prokaryotic biomass samples were taken at the continental rise/in abyssal plains that contribute 89% to the global ocean floor area^42^. Benthic prokaryotic densities were sampled to 39% in near-shore areas, to 8% at continental shelves, to 31% at continental slopes, and to 22% at the continental rise/in abyssal plains (Fig. 6). Benthic Bacteria and Archaea density samples were mostly taken at >200 m water depth (i.e., continental slope: 34 and 31%, respectively; continental rise and abyssal plains: 45 and 50%, respectively).

About half of the benthic prokaryotic biomass (52%) and two-third of the density (67%) records were surface sediment records. 32% (benthic prokaryotic biomass) to 48% (benthic prokaryotic density) of these surface sediment layers stretched from 0 to 1 cm. The thinnest sediment layers had thicknesses of 0.3 cm and the thickest sediment layers reached to 20 cm below the sediment surface.

## Data Availability

The R code used to generate Figs. 4, 5, and 6 can be found in *Zenodo*^43^.
